# Thalidomide exerts protective immunomodulatory action during *Klebsiella pneumoniae *B5055-induced acute lung infection in BALB/c mice

**DOI:** 10.1186/cc14008

**Published:** 2014-12-03

**Authors:** V Kumar, S Chhibber

**Affiliations:** 1 Department of Microbiology, Panjab University, Chandigarh, India

## Introduction

Thalidomide (α-naphthylimidoglutarimide), a psychoactive drug that readily crosses the blood-brain barrier, has been shown to exhibit anti-inflammatory, anti-angiogenic, immunomodulatory properties through a mechanism that is not fully established. Keeping these properties in mind, we have tried to find out the anti-inflammatory and immunomodulatory properties of thalidomide in mouse model of acute inflammation by introducing *Klebsiella pneumoniae *B5055 in BALB/c mice via the intranasal route.

## Methods

Acute lung infection (ALI) or pneumonia in BALB/c mice was induced via instillation of selected dose (10^4 ^CFU/ml) of bacteria (that is, *K. pneumoniae *B5055) intranasally. Mice were observed for 7 days and lungs were isolated on designated days for studying difference in bacterial load and other proinflammatory mediators using standard biochemical methods and ELISA.

## Results

The intranasal instillation of bacteria in this mouse model of acute pneumonia-induced inflammation led to significant increase in neutrophil infiltration into the lungs. This was further accompanied by an increased production of proinflammatory cytokines (that is, TNFα and IL-1α) and other mediators of inflammation (that is, malondialdehyde (MDA), myeloperoxidase (MPO) and nitric oxide (NO)) in the lung tissue. The animals, which received thalidomide alone orally or in combination with augmentin, 30 minutes prior to bacterial instillation into the lungs via intranasal route, showed significant (*P *≤ 0.05) decrease in neutrophil influx into the lungs. A significant (*P *≤ 0.05) decrease in the production of proinflammatory cytokines (that is, TNFα and IL-1α) and other biochemical mediators of acute inflammation (that is, MDA, MPO, and NO) was also observed in this group. But the augmentin treatment alone did not decrease these proinflammatory mediators significantly (*P *≥0.05) as compared to the control group.

## Conclusion

We therefore conclude that thalidomide ameliorates lung inflammation induced by *K. pneumoniae *B5055 without significantly (*P *≤ 0.05) decreasing the bacterial load in the lung tissue whereas augmentin takes care of bacterial proliferation. Hence, it can be used as an adjunct therapy along with antibiotics as an anti-inflammatory or an immunomodulatory agent in case of acute lung infection or pneumonia.

